# Comparison of microstructural evolution differences during dry-jet wet spinning and wet spinning for polyacrylonitrile precursor fiber

**DOI:** 10.1038/s41598-025-05449-4

**Published:** 2025-07-25

**Authors:** Jiping Wang, Xiaoqiang Ji, Xingfeng Liao, Yang Chen, Hongyan Song, Weisong Zhang

**Affiliations:** 1 School of Chemical Engineering and Materials, Changzhou Institute of Technology, Carbon Fiber New Materials Industry College, Changzhou, 213032 China; 2 Newtech Group Co., Ltd, Changzhou, 213032 China

**Keywords:** Polyacrylonitrile precursor fiber, Spinning method, Structure evolution, Polymers, Polymer characterization

## Abstract

This study compared the microstructural evolution of polyacrylonitrile (PAN) fibers, which serve as precursors for carbon fibers, during both dry-jet wet spinning and wet spinning processes. Detailed analyses were performed on fibers at various key stages of these two spinning methods using a range of techniques: scanning electron microscopy (SEM), atomic force microscopy (AFM), X-ray diffraction (XRD), wide-angle X-ray scattering (WAXS), and small-angle X-ray scattering (SAXS) and so forth. The main differences between the two schemes lie in the coagulation bath stage. After coagulations dry-jet wet spinning fibers display higher crystallinity (about 75%, compared to 55% for wet spinning), distinct yield behavior, and strain hardening during the tensile test. In addition, according to SAXS scattering patterns, pores in dry-jet wet spinning are mainly formed during the coagulation process, while in wet spinning, they are more likely to originate from subsequent stretching. However, qualitatively speaking, if we ignore the morphological differences of PAN precursor fibers from those two methods, as the spinning process progresses, the above differences gradually decrease. These findings provide novel insights and valuable guidance for optimizing the PAN fiber spinning process.

## Introduction

Carbon fibers, particularly those derived from polyacrylonitrile (PAN) precursors, have become a vital material in modern industrial and daily applications, such as spanning aerospace, automotive, energy, and sports equipment^1,2^. This is attributable to their exceptional mechanical properties, lightweight nature, and resistance to extreme environmental conditions. The manufacturing of PAN-based carbon fibers primarily entails polymerization, fiber spinning, stabilization, and carbonization^3,4^. Numerous studies have revealed that the multi-layer structure of carbon fibers^5–8^including the orientation and crystal size of graphite, the distribution and shape of micropores, the skin-core structure, and other factors, significantly impact the tensile strength and modulus of carbon fibers. These structural factors are directly associated with the microstructure of PAN precursor fibers formed during the spinning process^9,10^.

For PAN spinning, the strong polar interactions between acrylonitrile units result in a viscous flow temperature significantly higher than its degradation temperature^11^. Therefore, PAN precursors are primarily produced through solvent spinning. Filaments undergo coagulation, water washing, boiling water stretching, oiling, drying, steam stretching, and heat setting before reaching the final winder, with different total drawing ratios applied for each technique^5,12,13^. During the entire spinning process, several fundamental polymer physics processes occur simultaneously or sequentially, such as phase separation, polymer stress-induced crystallization accompanied by conformation stretching and relaxation during coagulation^14^cold drawing during steam stretching and so forth^15^. The structural characteristics after each step can vary significantly, with the formation of structure within each step closely related to the previous thermal history for the viscoelastic characteristic of polymers^16,17^. In industrial production, wet spinning and dry-jet wet spinning are the commonly adopted techniques for PAN precursor production^18^. The primary distinction between these two techniques is that in dry-jet wet spinning, the PAN solvent filaments pass through an air gap before entering the coagulation bath, where partial gelation and significant stretching occur^19^. Generally, dry-jet wet spinning led to greater structural orientation and better tensile properties, while wet spinning produced better interfacial adhesion between the fiber and matrix^12,20^.

Large amounts of work have been performed to investigate structure evolution during those two schemes respectively. Moskowitz^21^ investigated the influences of the stretching ratio during coagulation for wet spinning on PAN fiber morphology and thermal properties. Their work suggested that coagulation not only influences the initial structure and dictates orientation but also the structural evolution during the subsequent stretching stages. Yuhui Ao^22^ systematically studied the structural characteristics at key stages of the wet spinning process. They observed that the as-spun fibers exhibit a homogeneous 3D fibrillar network after coagulation, which may have significant influences on the subsequent stretching, orientation, and crystallization. Liu^9^ systematically investigated the effects of different spinning methods on the structures and performances of polyacrylonitrile fibers, especially the structural evolution during stabilization and carbonization. In conclusion, the initial scheme differences between those two techniques dramatically determined the structure evolution of PAN fibers during the entire spinning processes^23^. To date, the fundamental understanding about microstructural evolution mechanism remains insufficiently, particularly regarding the molecular-level. Therefore, a systematic investigation of microstructural evolution throughout successive processing stages in both methodologies is crucial for optimizing the PAN fiber spinning process.

**Fig. 1:**
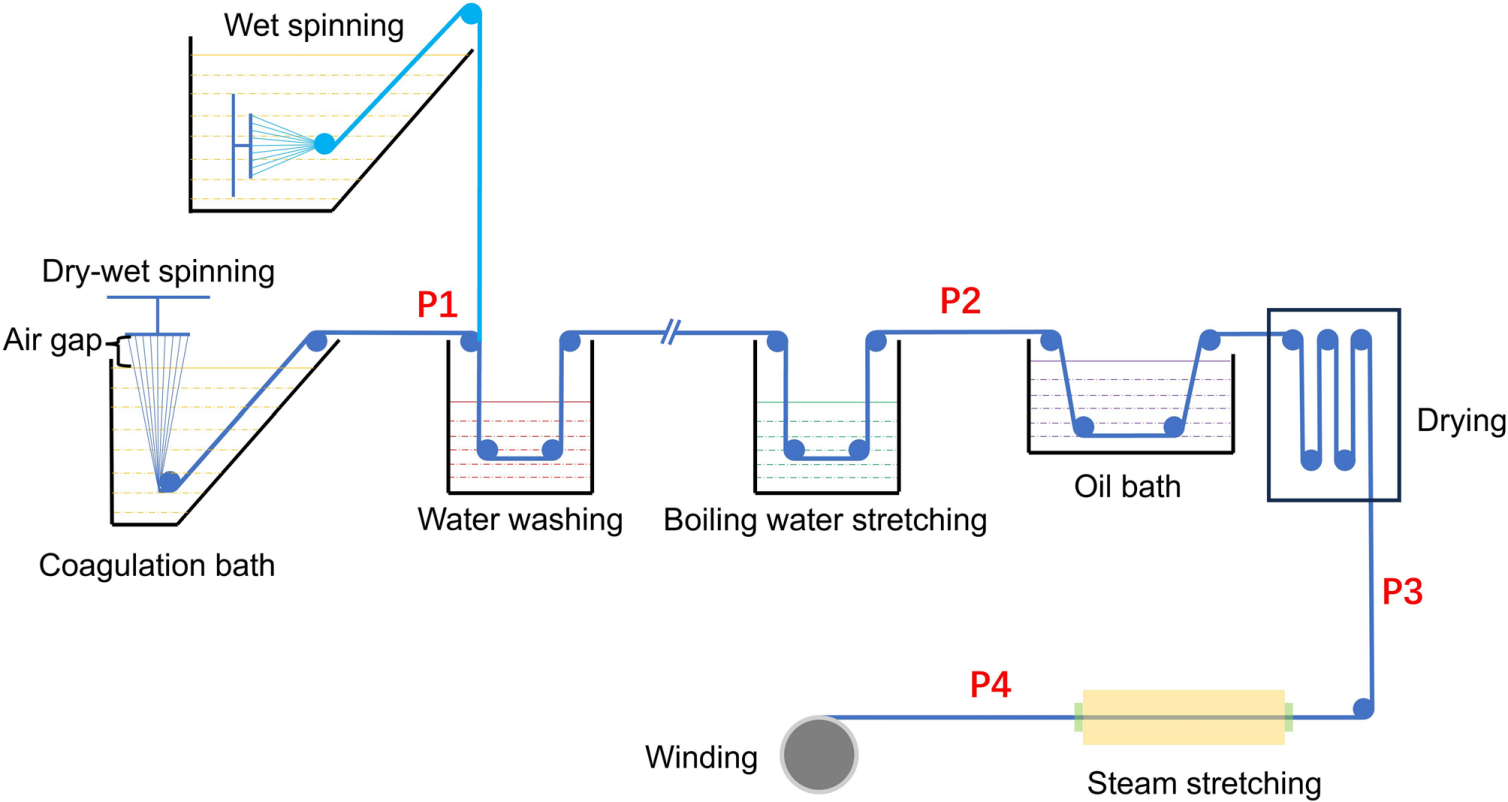
Schematics of dry-jet wet spinning as well as wet spinning post processes.

While existing studies predominantly focus on single methodology analyses, here we present a systematic comparison of microstructural transformations between wet spinning and dry-jet wet spinning throughout the whole spinning processes. Fibers were randomly selected from each spinning stage of both a wet spinning line and a dry-jet wet spinning line, details about how we performed the spinning as well as sample collections are given in the following section. Fibers were chosen from four key stages for each process, as depicted in Fig. 1: P1 represents fibers exiting the coagulation bath; P2 denotes samples after water washing; P3 indicates the dried samples; and P4 represents fibers after steam stretching. The fibers’ morphology, crystalline structure, and micropore structure were investigated using 2D wide/small-angle X-ray scattering (WAXS and SAXS), X-ray diffraction (XRD), scanning electron microscopy, and atomic force microscopy (AFM). Additionally, the strength of the fibers was measured using a single-fiber tensile tester. We discussed the differences in microstructural evolution between dry-jet wet spinning and wet spinning in detail and constructed the structure-property relationship for fibers at different stages. Furthermore, we try to give a molecular level scenario about the differences between those two spinning methods from polymer physics and chain dynamics point of view. Interestingly, although a great many differences could be observed in terms of the crystal structure, pore structure, and tensile testing results of the fibers emerging from the coagulation bath obtained through different schemes (P1 samples), as the spinning process progresses, those differences gradually diminish, provided that we overlook the morphological disparities of the PAN precursor fibers. This work would be valuable for researchers and manufacturers to better understand the intrinsic mechanisms of various spinning technologies.

## Experimental section

### Material and sample preparation

Fibers at each stage were sampled at the end of the coagulation bath, water washing, drying, and steam stretching. They were kindly offer by Newtry Carbon Valley Co., Ltd., from an experimental dry-jet wet spinning and a wet spinning line respectively. The samples were labeled as WP1 to WP4 for wet spinning and DP1 to DP4 for dry-jet wet spinning. All samples were air-dried before testing.

The details about the spinning processes are as following. The chemical precursor used in this study was homogeneously polymerized polyacrylonitrile (PAN) in dimethyl sulfoxide (DMSO). The parameters employed in this experiment align with those commonly adopted in conventional carbon fiber spinning processes^24^. Specifically, regarding spinneret parameters, significant differences exist between dry-jet wet spinning and wet spinning due to variations in processing conditions and spinning speeds. For wet spinning, the spinneret orifice diameter is 50 μm, whereas for dry-jet wet spinning, it is 150 μm. In the wet spinning coagulation bath, the temperature is typically maintained at 60 °C, while in dry-jet wet spinning, the coagulation bath temperature is much lower, approximately 20 °C. During the washing process, the temperature profiles for both dry-jet wet spinning and wet spinning are similar, starting from the primary washing stage and gradually increasing. The average temperature in the initial stages is around 70 °C, with the final stage reaching 95 °C. For both processes, the drying and densification temperature is 160 °C, and the steam stretching temperature is 170 °C. The ambient air temperature throughout the spinning process remains stable at 20 °C. These parameters are generally consistent with data reported in the literature.

To eliminate the effects of orientation, fibers are cut into short segment within 1 mm through a specialized fiber cutting scissors, following those short segments are grinded with an agate mortar carefully. For 2D SAXS and WAXS testing, the fibers were aligned in a bundle with lengths exceeding 30 mm and diameters around 3 mm.

### Characterization

The surface morphologies and cross-sections of the fibers were characterized using a scanning electron microscope (SEM) (JSM-7800 F, JEOL Ltd., Japan) operated at an accelerated voltage of 5 kV, after sputter-coating with a thin platinum layer. Fiber diameters were measured from SEM images, with 30 specimens tested for each sample. An atomic force microscope (AFM) (Dimension Fast Scan, Bruker, Germany) was employed to examine the roughness of the fiber surface, with a scanning area of 4 μm × 4 μm. Variations of crystallinity and size of crystals of PAN fibers during the spinning process were determined using an X-ray diffractometer (D8 ADVANCE, Bruker, Germany) operating at 40 kV and 40 mA with Cu Kα radiation (wavelength λ = 0.1542 nm). 2D SAXS and WAXS were performed on Xenocs xeuss 2.0 equipped with a Pilatus 3 S 2 M X-ray detector (with a pixel size equal to 175 μm), and the collected data were analyzed using Fit2D V12.052 developed by ESRF. Tensible tests were performed through an electronic single fiber strength machine (Wenzhou Fangyuan instrument CO., LTD.) with a strain rate of 20 mm/min. Thermal analysis was conducted using a Mettler Toledo DSC1 differential scanning calorimeter equipped with a refrigerant compressor (TC100 MT Intracooler) under nitrogen atmosphere. The temperature program was set from − 20 to 300 °C at a heating rate of 5 K/min. For surface chemical structure characterization of PAN precursor fibers from both techniques, ATR-FTIR (Nicolet iS 10 with an ATR attachment, 32 scans, 4 cm⁻¹ resolution) and confocal Raman spectroscopy (Horiba LabRAM HR Evolution, 532 nm laser source, 50× lens) were employed.

## Result and discussion

### Surface morphology of PAN fibers

**Fig. 2:**
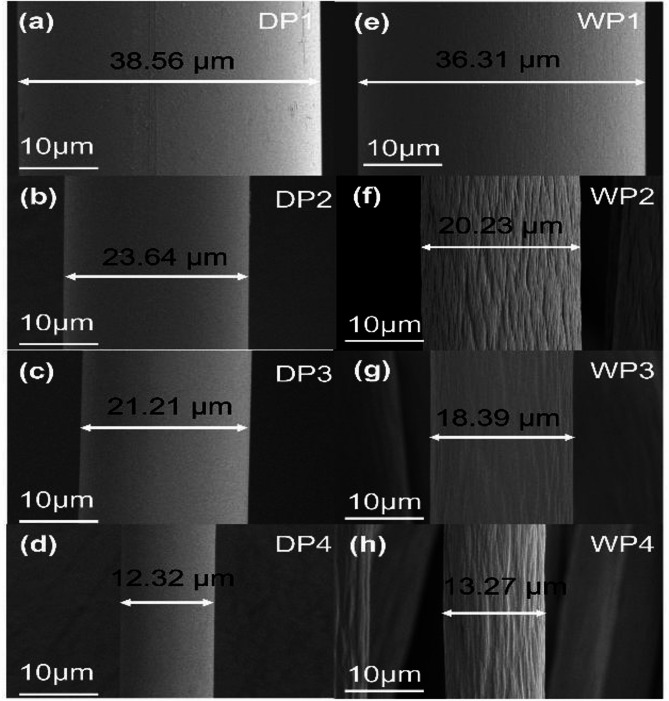
SEM images of PAN fibers surface morphologies evolution for each post-spinning processing stage. Figures (**a–d**) are from dry-jet wet scheme and figures (**e–h**) are from wet scheme.

**Fig. 3:**
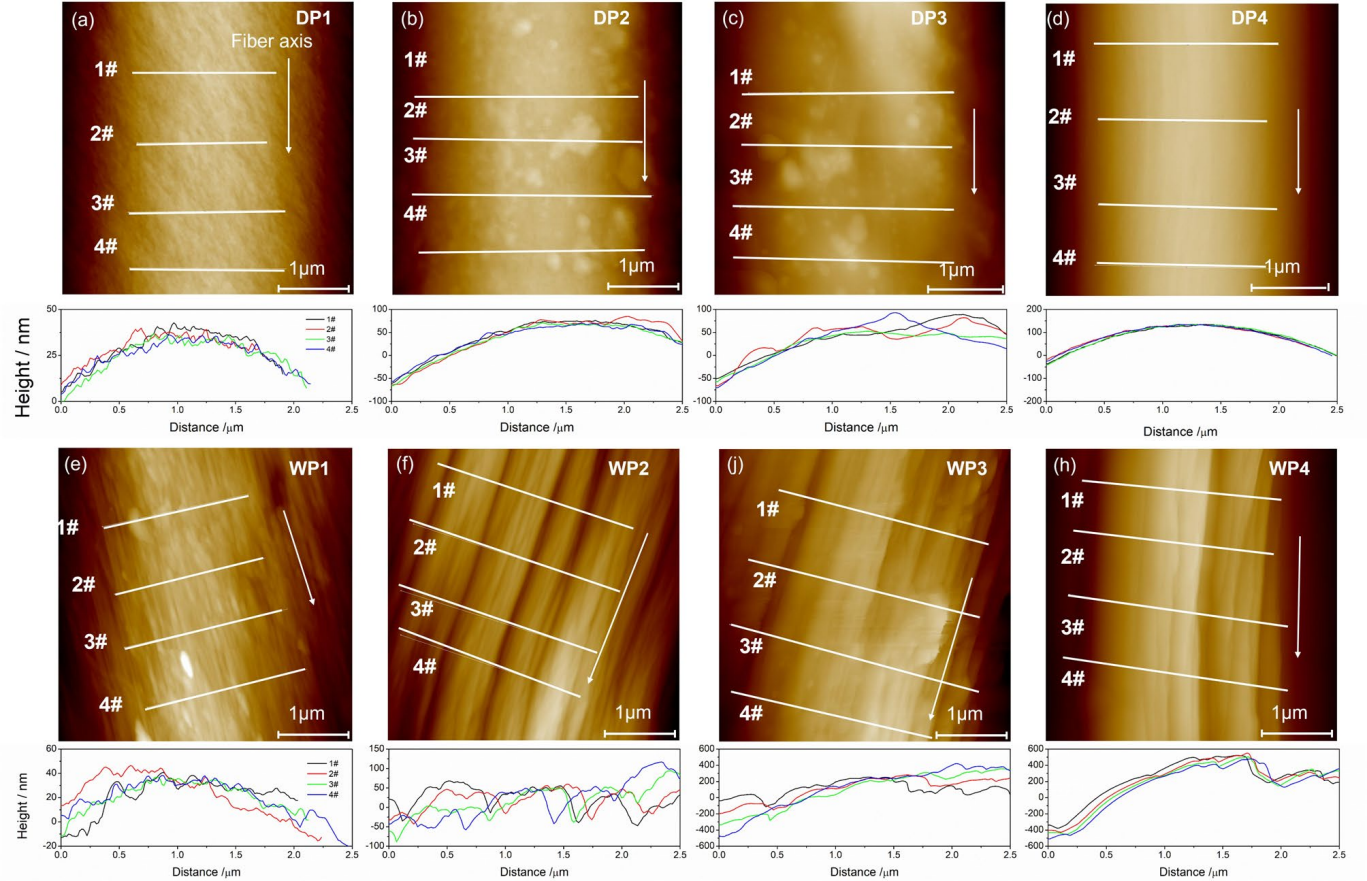
AFM images of fiber surface fluctuation as well as the variation of height. Figure (**a–d**) represents dry-jet wet spinning technology and (**e–h**) are from the wet spinning method.

SEM and AFM techniques were applied for the morphology testing as shown in Figs. 2 and 3 respectively. Figure 2a–d gives the fiber morphologies evolution during dry-jet wet spinning processes, and Fig. 2e–h represents the morphologies variation for wet spinning. The dramatic difference lies in the totally different surface textures between dry-jet wet and wet spinning. For each stage of dry-jet wet spinning, the fiber surface seems smoother compared with the wet-spinning technique. It is owing that before entering into the coagulation bath, filaments undergo a partial gelation within the air gap for dry-jet wet spinning, as illustrated in Fig. 1. In addition, the diameter of fibers after coagulation is almost the same, 38.56 μm for dry-jet wet spinning and 36.31 μm for wet spinning, as shown in Fig. 2a and e. However, the diameter of the spinneret orifice for dry-jet wet spinning is usually several times larger than that of wet spinning. Therefore, the similar diameter of the fiber after coagulation suggested dry-jet wet scheme could offer a much larger stretching ratio for the filaments. The orientation of chain conformation during dry-jet wet spinning should be much larger than that of wet stretching, this speculation is in agreement with following WAXS and SAXS data.

Figure 3 offers a comparative examination of the surface roughness progression in PAN (polyacrylonitrile) fibers throughout both dry-jet wet and wet-spinning procedures. The AFM height images, depicted in Fig. 3a–d, illustrate the various phases of the dry-jet wet spinning process, while Fig. 3e–h represent the stages of the wet spinning process. Four lines were drawn perpendicular to the fiber axis in each AFM image, serving as benchmarks for measuring height variations along the fiber surface. The graphs beneath each AFM image present the height variation data derived from these lines, offering a quantitative portrayal of surface roughness at each spinning stage.

Upon coagulation, the height fluctuation of the fibrous surface is nearly identical for both methods, as evidenced in Fig. 3a and e, with fluctuations around tens of nanometers. Following water washing, the surface morphology of dry-jet wet spun fibers becomes smoother, whereas wet-spun fibers exhibit increased roughness, with height fluctuations expanding to approximately 100 nm. This discrepancy may be attributed to high rates of diffusion during water washing which may induce stronger surface phase separation for the wet spinning method. However, for the dry-jet wet spinning process, owing to the surface gelation formed before entering the coagulation bath, double diffusion during the water-washing step is much more difficult to damage surface morphology. During the drying process, the evaporation of water within the fibers further damages the surface layer, leading to a renewed increase in surface height fluctuations. Notably, the increase in surface fluctuation for wet spinning is significantly more pronounced than that for dry-jet wet spinning. Finally, steam stretching substantially densifies the fibers, as demonstrated in the SEM image in Fig. 2 above, where the fiber diameter undergoes a significant reduction after steam stretching. For both methods, steam stretching results in a smoother surface compared to previous stages. In wet spinning, steam stretching cannot eliminate all fluctuations but rather smooth out short-term variations, which infer that small-scale height fluctuations perpendicular to the fiber axis merge into larger ones. Conversely, in the dry-jet wet spinning technique, both small-scale and large-scale height fluctuations are eliminated during the steam stretching process.

### Evolution of crystal structure during spinning

**Fig. 4:**
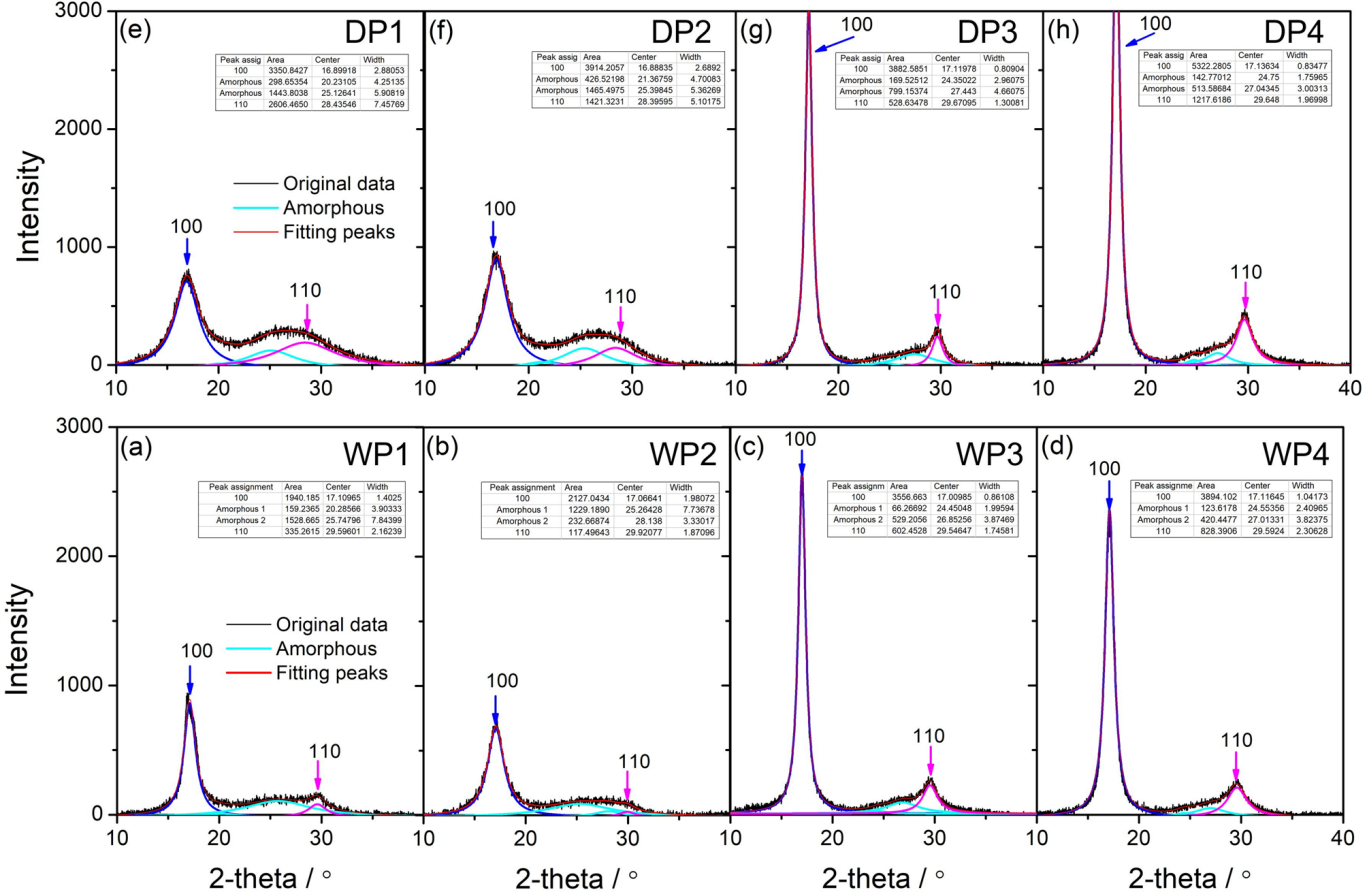
1D-XRD data of fibers from different methods as the spinning progressive, dry-jet wet method for (**a–d**) and wet method for (**e–h**). Black lines give the origin data, and the thin lines represent the fitting curves. Insert tables give the details of fitting results including peak position, widths as well as the areas.

Figure 4 presents the powder X-ray diffraction data obtained from each stage of spinning for both dry-jet wet spinning (Fig. 4a–d) and wet spinning (Fig. 4e–h). The black curves represent the original XRD data collected during our testing. To meticulously analyze the evolution of crystallinity and crystal structure throughout the spinning process, we further fitted the curves using four Lorentz peaks. As depicted in Fig. 4, we have identified two broad amorphous peaks, centered at 24° and 27°, respectively. Additionally, the peaks local at 17° and 29° are attributed to the characteristic 100 and 110 planes of the PAN crystal. The insert tables in each figure give fitting results. Qualitatively speaking, as the spinning progresses, crystal signals become more and more obvious, which is associated with stretching-induced polymer crystallization. In addition, compared with wet spinning, during the dry-jet wet spinning process especially before drying the signal of the 110 planes is much sharper, which infers that the crystals acquired from the dry-jet wet scheme are much more perfect.

**Fig. 5:**
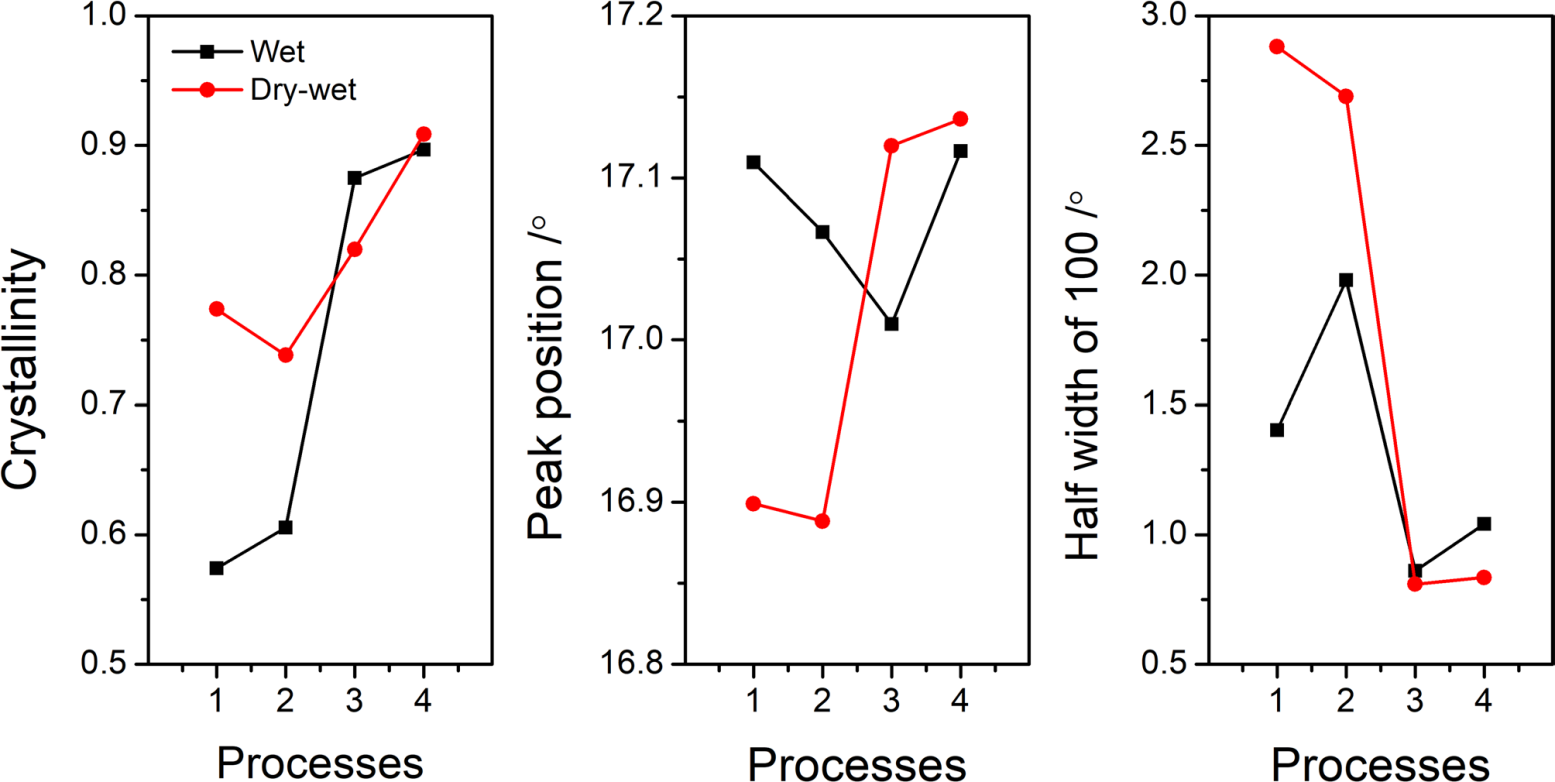
Evolution of crystallinity (**a**), peak position (**b**), and half-width (**c**) of 100. Data is acquired from the tables in above Fig. 4.

Furthermore, Fig. 5 illustrates the progression of crystallinity (Fig. 5a), the peak position of the 100 planes in the PAN crystal (Fig. 5b), and the half-width of the 100 planes (Fig. 5c) as the spinning process evolves for both wet and dry-jet wet spinning techniques. Crystallinity, quantified as the ratio of crystal peak areas to the total area under the curve, is derived from the peak fitting data presented in Fig. 4. Notably, Fig. 5a highlights a striking disparity between the two methods: at the onset of spinning, the crystallinity achieved through the dry-jet wet spinning method is substantially higher than that of the wet spinning method. This disparity suggests that during dry-jet wet spinning, the filaments experience a more pronounced stretching-induced crystallization, which is further corroborated by subsequent Wide-Angle X-ray Scattering (WAXS) data. Moreover, the decrease in crystallinity observed after water washing in the dry-jet wet spinning process, as depicted in Fig. 5a, may be attributed to stretching-induced melting-recrystallization. In contrast, for the wet spinning method, we observed a slight increase in crystallinity at the same stage. This difference arises because the wet spinning method is unable to achieve as strong a stretch in the coagulation bath compared to dry-jet wet spinning. As a result, stretching-induced polymer crystallization in the wet spinning method primarily initiates during the water-washing stage. A more detailed analysis of this phenomenon will be provided based on the following WAXS results. Additionally, the shifts in the X-ray Diffraction (XRD) peak position reflect alterations in the crystal plane during spinning, with a larger 2-theta angle indicating a denser lattice structure. The half-width of the 100 peaks is related to the extension size of the 100 planes. According to the Scherrer equation^25,26^, a smaller half-width signifies a larger crystal size. As demonstrated in Fig. 5b and c, the dry-jet wet spinning process exhibits more significant changes in both the peak position and half-width of 100 planes compared to the wet spinning method. This observation may imply that during dry-jet wet spinning, the PAN crystal undergoes a more intense melting or damage reorganization process. This reorganization process ultimately leads to a more perfection of the crystal structure of final PAN precursor fibers.

### Evolution of crystal orientation during spinning

**Fig. 6:**
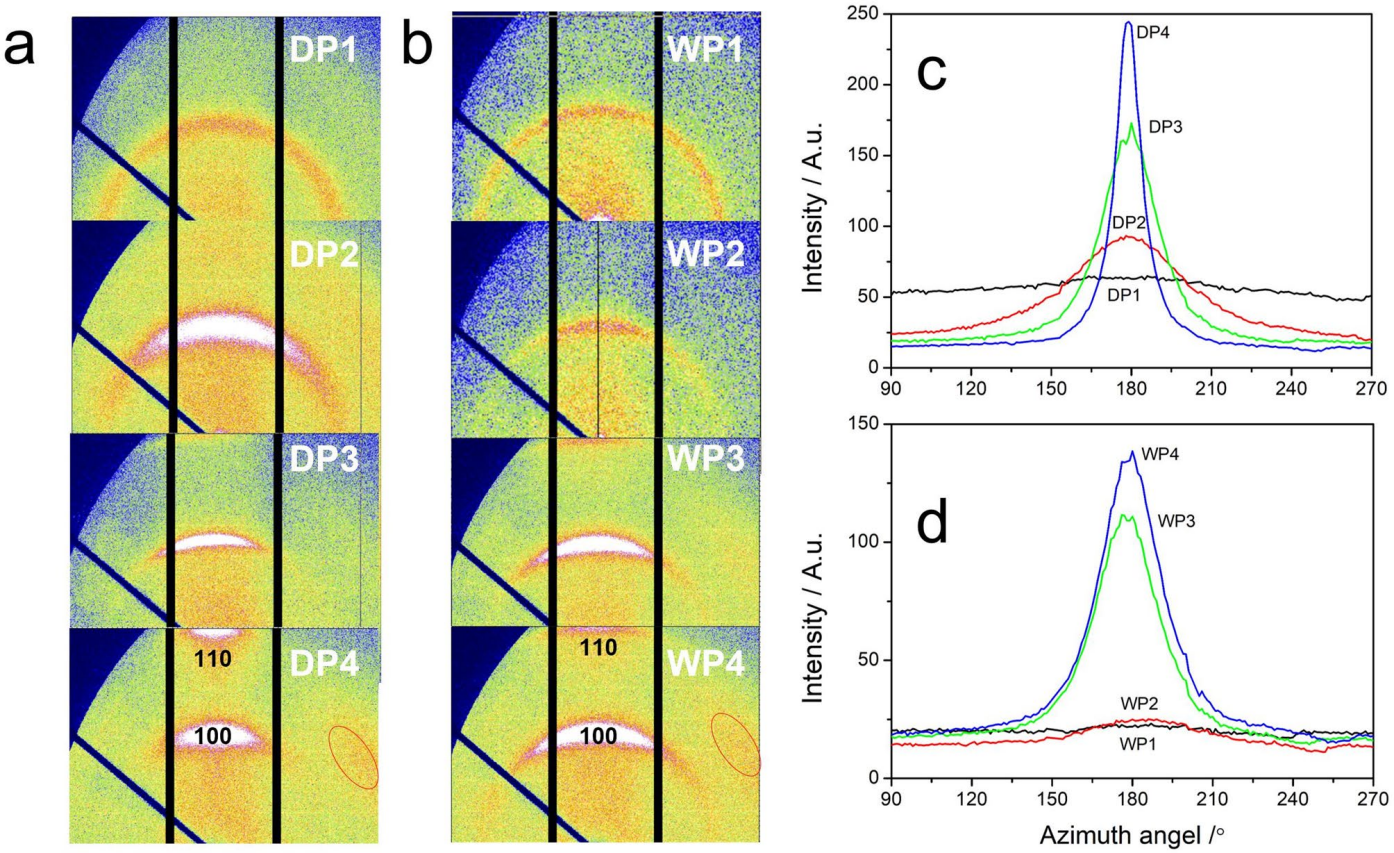
WAXS patterns of PAN fibers acquired from dry-jet wet method (**a**) and wet method (**b**). Figures (**c**,**d**) are the integration of the azimuth of the pattern shown in a and b respectively. The white arrows give the fiber alignment direction.

Figure 6 presents the evolution of WAXS patterns at each stage for both dry-jet wet spinning (Fig. 6a) and wet spinning (Fig. 6b), along with the integrated intensity distribution within the azimuth angle range from 90° to 270° for the patterns displayed in Fig. 6a and b. Generally, as the spinning process progresses, the scattering signals become increasingly anisotropic for both methods, indicating a gradual improvement in crystal orientation. Additionally, a signal appearing almost 45° from the equator was observed after stream stretching in both cases, as highlighted by the red circles in Fig. 6a-DP4 and 6b-WP4. Given that the scattering patterns are centrally symmetric, this pattern represents a four-point scattering signal. It arises from the twisting of lamellae attached to the highly extended fibers within a moderately stretched field^27,28^. This also suggests that shish-kebab crystals are primarily formed during the steam stretching stage for both spinning methods^29^.

There are two notable differences between the two methods. Firstly, in the initial two stages of dry-jet wet spinning (coagulation and water washing), significant crystal orientation was already observed, as demonstrated in Fig. 6c. The azimuthal integration of scattering intensity for DP1 and DP2 samples exhibited a distinct peak at 180°, indicating the occurring of crystal orientation. By contrast, WP1 and WP2 samples showed no obvious intensity fluctuations in their azimuthal integral curves during the same stages (Fig. 6d), suggesting a lack of pronounced crystallographic orientation at this stage of processing. In addition, the dry-jet wet spinning process exhibits a significantly higher final crystal orientation after steam stretching compared to wet spinning, as evidenced by the data presented in Fig. 6a-DP4 and 6b-WP4, along with the corresponding much sharper peak shown in Fig. 6c-DP4 comparing with Fig. 6d-WP4. Therefore, companying with above XRD data, we could draw a conclusion that during the dry-jet wet spinning process polymer experiences a much stronger conformational stretching comparing with wet spinning, which is responsible for the higher crystallinity as well as crystal orientation. Its microscale origin will be discussed in the following sections.

### Evolution of pore structure during spinning

**Fig. 7:**
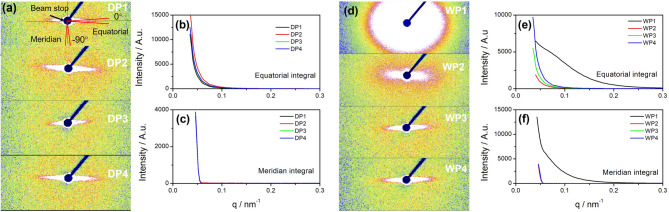
SAXS patterns of PAN fibers acquired from dry-jet wet method (**a**) and wet method (**d**). Figures (**b**,**c**) are variations of 1D SAXS intensity of (**a**) integrating along equatorial and meridian directions respectively. Figures (**e**,**f**) are integrated from (**d**). Schematics of equatorial and meridian directions are illustrated in Figure a-DP1.

Pore structure represents the primary defect contributing to carbon fiber failure, highlighting the importance of elucidating the pore evolution mechanism during the spinning process. In this study, small-angle X-ray scattering (SAXS) was employed to characterize the pore structure features. Figure 7a and d illustrate the SAXS pattern evolution as spinning progresses for dry-jet wet and wet-spinning methods, respectively. Furthermore, Fig. 7b and c, as well as 7e and 7f, present the integrated intensity versus *q* values derived from Fig. 7a and d. Specifically, Fig. 7b and e depict intensity variations along the equatorial direction, while Fig. 7c and f display those along the meridian direction, as labeled in each respective figure. The integration specifics are outlined in the pattern depicted in Fig. 7a-DP1, with the azimuthal angle range spanning from − 2° to 2° for the equatorial integration and from − 92° to − 88° for the meridian integration.

As observed, the scattering pattern’s shape underwent subtle variations as spinning progressed in the dry-jet wet spinning method. The needle-like signal indicated the formation of ellipsoidal void structures within the fibers along the stretching direction during the initial stages of dry-jet wet spinning. The intensity variations along the equatorial direction, depicted in Fig. 7b, were primarily attributed to the variation of void size and size distribution at each spinning stage. Slower intensity decay rates implied a broader distribution of void sizes. Conversely, in the wet spinning method, the pattern remained nearly isotropic after coagulation, suggesting that the filaments within the coagulation bath did not undergo sufficient elongation. Furthermore, both the equatorial and meridian integration data, shown in Fig. 7e and f, exhibited a broad peak signal, marked by a red arrow in each figure. This observation suggests the presence of a periodic structure with a long period of approximately tens of nanometers. There are two potential explanations: either the period is related to the PAN crystal lamellar structure formed during coagulation^30^or it reflects phase separation. Further details will be explored in our subsequent work.

**Fig. 8:**
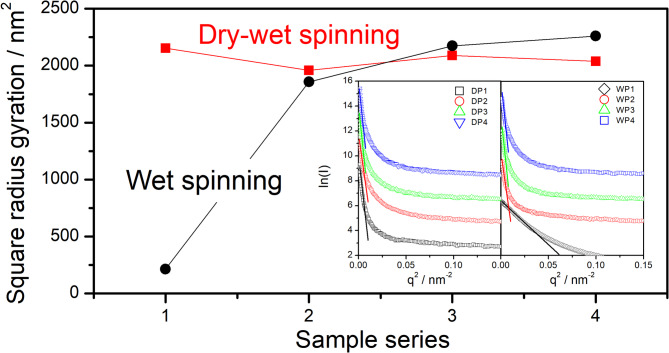
Variation of mean square radius gyration of pores for different methods as spinning progressive. The insets give Guinier plots of scattering intensity data derived from Fig. 7b and e.

Figure 8 illustrates the progression of the square radius of gyration of pores throughout the spinning processes for various methods. The inset figures display Guinier plots (ln(I) versus q^2^) derived from the data presented in Fig. 7b and e^31^. The square radius of gyration is determined from the slope of the linear fitting within the low q² range, as indicated by the thin lines in the insets. Guinier’s theorem is as follows:

$$\:\text{ln}\left(I\right)\propto\:-\frac{1}{3}{R}_{G}^{2}{q}^{2}$$

where *I* is the scattering intensity, $\:{R}_{G}^{2}$ is the square radius gyration. As one can see the pore size fluctuation for dry-jet wet spinning is much smoother compared with wet spinning. It is inferred that the pore structure is mainly formed during coagulation for the dry-jet wet spinning method, however for the wet spinning method, the pore structure comes from further stretching after coagulation. In addition, the Guinier plot of WP1 shows a much wider linear region compared with other samples, which suggests a much homogeneous and sphere-like density fluctuation structure is formed after coagulation during wet spinning. Considering that negative drafting is always performed in the coagulation bath for the wet spinning method, as well as the above WAXS and SAXS data also suggesting that conformation stretching is not that obvious, thus such a homogeneous sphere-like phase separation structure may be caused by the interplay between phase separation coming from variation of temperature and concentration and crystallization of PAN. In addition, due to the larger pixel size (175 μm as mentioned in above Sect. 2.2), a key deficiency arises: it hinders precise calculation of the azimuthal scattering intensity distribution within the q-value range corresponding to the pore sizes of interest—mainly the low-q range, especially along the meridian direction (corresponding to the pore long axis). This limitation makes detailed analysis about the pore size distribution is invalidation in present which will be discussed in our following work.

### Tensile performance of PAN fibers at each stage

**Fig. 9:**
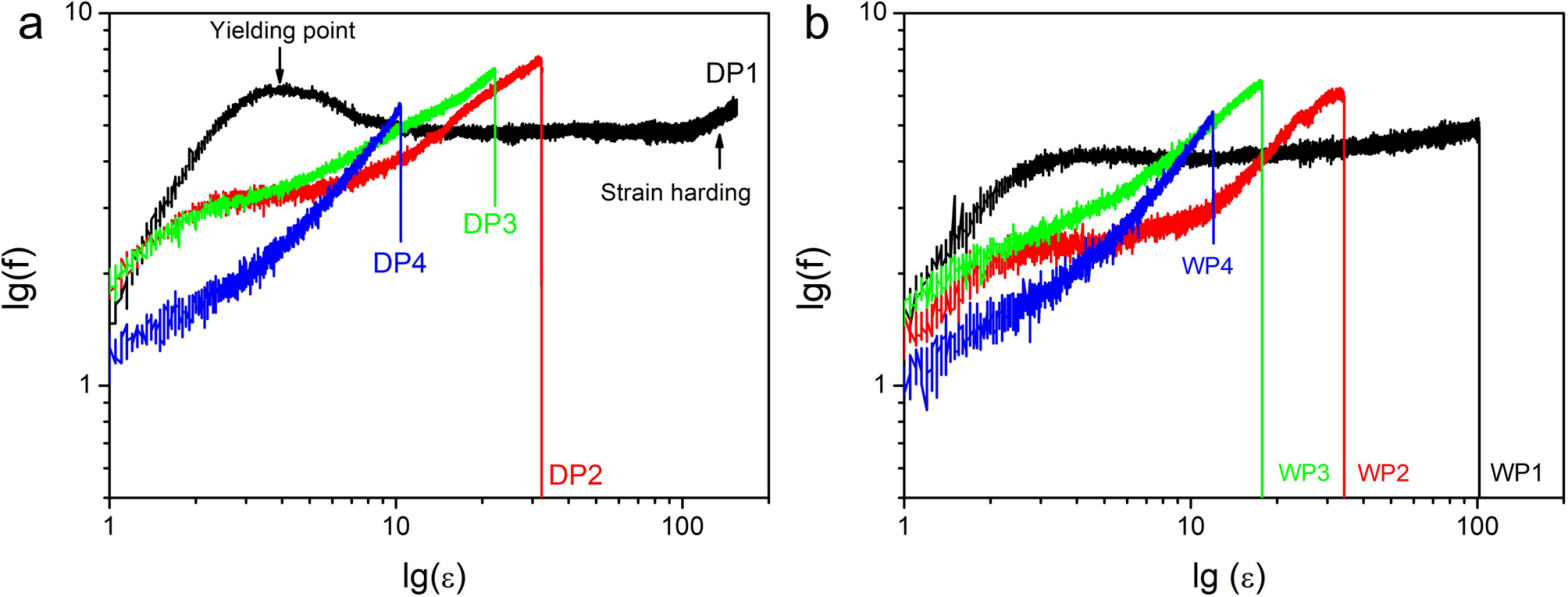
Tensile performance of PAN fibers from P1 to P4. Figure (**a**,** b**) give tensile force vs. strain curves in a double logarithmic coordinate for the dry-jet wet spinning method (**a**) and wet spinning method (**b**) respectively.

Figure 9 gives the tensile performance of fibers at each stage for dry-jet wet spinning (Fig. 9a) and wet spinning (Fig. 9b) methods respectively. A double logarithmic coordinate was employed to better display data under low strain conditions as well as the yield point. The biggest differences lie in the earliest stage of spinning. As one can see both DP1 and WP1 exhibited large plastic deformations. For WP1 no strain hardening could be observed during the whole deformation process, in addition, the yield behavior is not very obvious. However, for DP1 an obvious yielding after elastic deformation, as well as a strain hardening onset around 100% strain could be observed clearly. In addition, DP1 didn’t snap within our equipment strain range, according to a previous report DP1 sample may hold above 200% strain at break^12^. Based on the above structural analysis, compared with the WP1 sample, the DP1 sample shows higher crystallinity and more significant crystal and chain conformation orientation along the spinning direction. This indicates that at the chain scale, the DP1 sample undergoes more obvious deformation along the spinning direction. The reason why dry-jet wet spinning can provide much stronger conformational stretching should be related to the air gap marked in Fig. 1. In this stage, the polymer is in a good solvent, so it can be stretched much faster. This fast stretching not only provides a much higher degree of conformational stretching but also facilitates the disentanglement of polymer chains^32–35^. Specifically, the more obvious yielding phenomenon observed in the DP1 sample (Fig. 9a) may originate from the rupture of lamellae along the stretching direction. This explanation also helps to clarify why the crystallinity of DP2 is lower than that of DP1 (Fig. 5a). It is hypothesized that the strain hardening shown in Fig. 9a arises from stretching-induced crystallization in the semi-crystalline polymer matrix. As shown, the relatively slow spinning rate in wet spinning is less likely to change the entanglement state in the filaments. During the tensile test below the glass transition temperature of PAN (about 60 °C), a high entanglement density may make the sample more brittle. In contrast, the relatively fast spinning rate in dry-jet wet spinning hinders the recovery of the entangled network, thereby facilitating disentanglement. Therefore, under large deformations, the chains in DP1 are more likely to reorganize into crystalline domains, which contributes to the observed strain hardening. Following from P2 to P4 for both schemes, the shape of strain-stress curves is almost the same at each stage, which inferred that during those spinning stages fibers may experience the same physical processes even though the dramatically difference at the beginning. In addition, in previous simulation works of Jiping Wang^36^one author of the present work predicted that strain may erase the previous thermal history. In this work, as shown in the above XRD, WAXS, and SAXS data as well as the stress-strain curves, from a qualitative point of view, even though the big differences at the early stage of spinning for those two methods, the final structure characteristic as well as the mechanical performance after steam stretching are almost the same. Therefore, our present work further confirmed that assumption to some degree.

### Thermal and surface analysis of PAN precursor fibers from two techniques

**Fig. 10:**
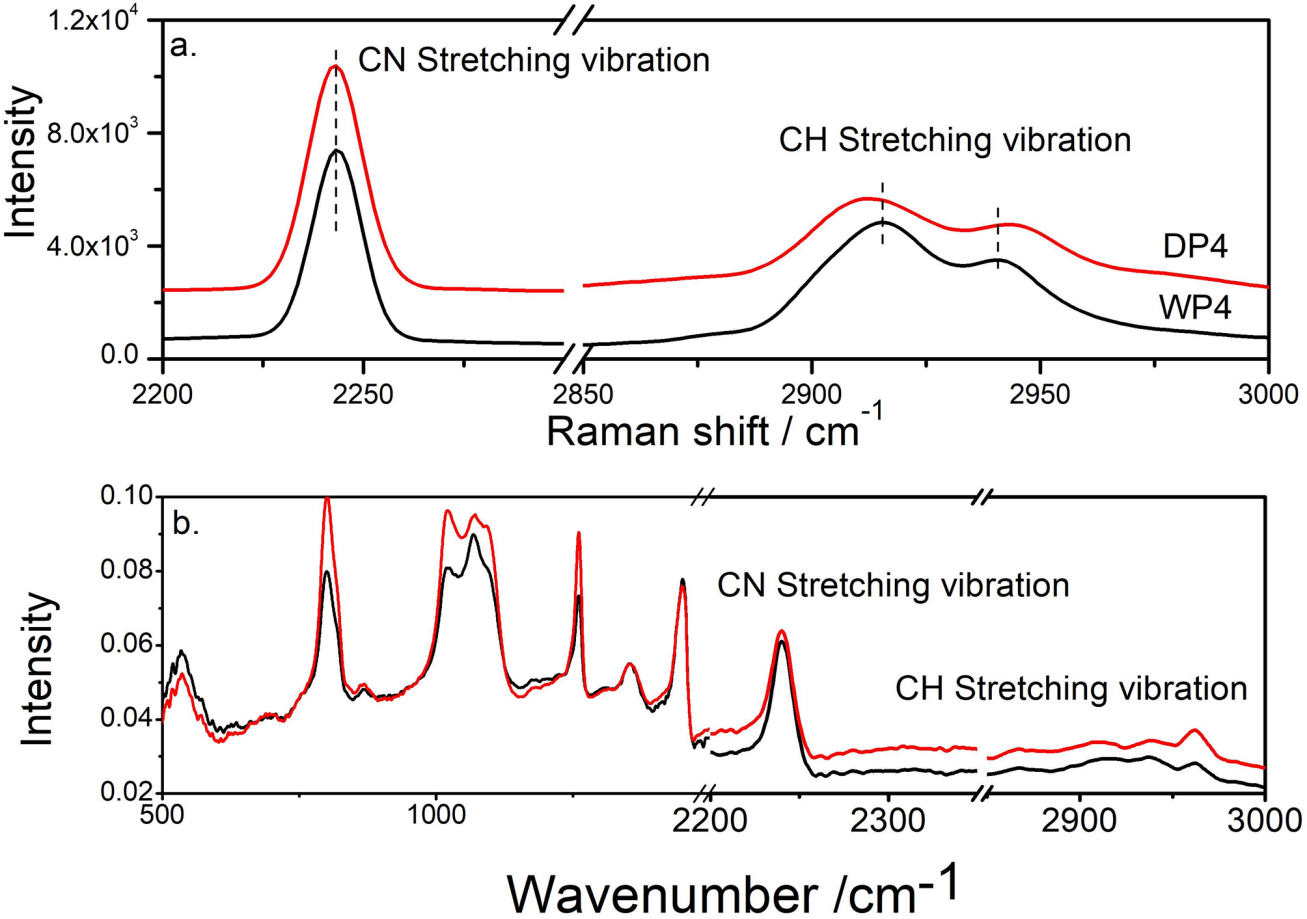
Raman (**a**) and FTIR (**b**) spectroscopy for PAN precursors from dry-jet wet spinning (DP4) and wet spinning (WP4).

Figure 10a and b present the Raman and FTIR test results for the WP4 and DP4 samples, with curves vertically shifted for better visualization. Band assignment are according to previous reports^37,38^. As shown, the main characteristic bands in both FTIR and Raman spectra are nearly identical for the two samples, indicating that their chemical compositions are almost the same.

**Fig. 11:**
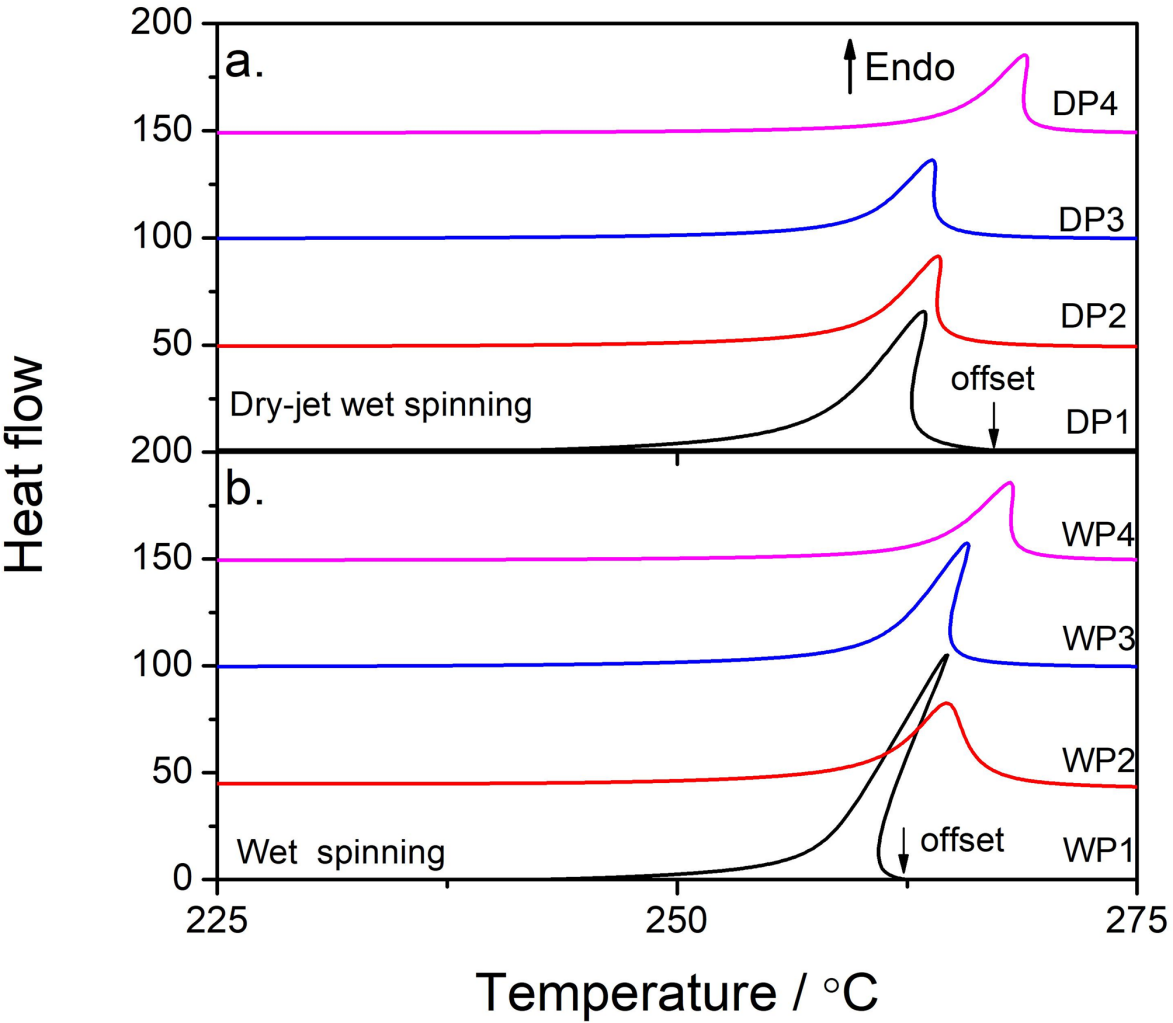
The DSC heating curves for samples prepared by the two spinning methods, as labeled on each curve. Figure (**a**) presents samples from the dry-jet wet spinning method, while Figure (**b**) shows curves from wet spinning. The black arrows indicate the offset temperatures of the endothermal peaks for the DP1 and WP1 samples, respectively.

Furthermore, Fig. 11a and b display the DSC heating curves for fibers at different stages of the dry-jet wet spinning and wet spinning methods. An obvious endothermal peak is observed in the temperature range of 225 °C to 375 °C. This peak does not correspond to the melting of PAN crystals but is associated with the cyclization of nitrile groups, which leads to the formation of a more heat-resistant trapezoidal structure^39^. Notably, samples DP4 and WP4 exhibit nearly identical thermal behaviors, with no significant differences in the onset temperature, peak temperature as well as offset temperature. In contrast, for DP1 and WP1, the endothermal peak of DP1 is much broader than that of WP1 (see the black arrows on DP1 and WP1, marking the offset temperatures: 262 °C for WP1 and 267 °C for DP1). This suggests that a larger amount of nitrile groups requires higher temperatures to induce cyclization. We attribute this to the higher crystallinity of DP1, which provides better thermal resistance (as shown in Fig. 5a). The DSC results further confirm our core hypothesis that strain may eliminate pre-existing structural differences.

## Conclusion

This paper compares dry-jet wet spinning and wet-spinning processes for polyacrylonitrile (PAN) fibers, focusing on morphology, crystal structure, crystal orientation, pore structure, tensile properties chemical compositions as well as the thermal properties. Using SEM, AFM, XRD, WAXS, SAXS, FTIR/Raman, and DSC. The study reveals several key differences. Fibers from both methods have similar diameters after coagulation, but dry-jet wet spinning always uses larger spinneret pores, which inferred a higher draw ratio at the early stage compared with wet-spinning. AFM confirms smoother surfaces in dry-jet wet spun fibers, especially after washing and drying. XRD and WAXS show more pronounced crystal formation and orientation in dry-jet wet spinning, with higher crystallinity and refined crystal structures. SAXS analysis indicates pores form mainly during coagulation in dry-jet wet spinning while wet-spinning pores derive more from stretching. Dry-jet wet spun fibers exhibit distinct yield behavior early in plastic deformation, and strain hardening before broken, which may be associated with the faster spinning rates promoting chain disentanglement. However, tensile properties converge as the process progresses. Overall, even though dry-jet wet spinning offers advantages in fiber surface, crystal structure, orientation, and pore structure, thermal properties especially initially. As the process deepens, fibers from both methods become similar from a polymer physics point of view. These findings provide some new insights and guidance for optimizing PAN fiber spinning.

## Data Availability

The datasets used and/or analysed during the current study available from the corresponding author on reasonable request.

## References

[1] Huang, Y. et al. Mussel-inspired adhesive and carbon fiber conductive hydrogel for flexible sensors. *ACS Appl. Polym. Mater.* **5**, 5707–5715. 10.1021/acsapm.3c00983 (2023).

[2] Chae, H. G. et al. High strength and high modulus carbon fibers. *Carbon* **93**, 81–87. 10.1016/j.carbon.2015.05.016 (2015).

[3] Li, D. H., Lu, C. X., Hao, J. J. & Wang, H. M. A comparative analysis of polyacrylonitrile-based carbon fibers: (I) microstructures. *Carbon* **176**, 1 (2021).

[4] Lu, J. et al. Microstructure and properties of polyacrylonitrile based carbon fibers. *Polym. Test.* **81**, 106267. 10.1016/j.polymertesting.2019.106267 (2020).

[5] Kim, D. W. et al. Effect of low processing rate on homogeneous microstructural evolution of polyacrylonitrile-based carbon fibers. *Carbon Lett.* **29**, 479–485. 10.1007/s42823-019-00047-7 (2019).

[6] Okuda, H. et al. Investigating nanostructures in carbon fibres using Raman spectroscopy. *Carbon* **130**, 178–184. 10.1016/j.carbon.2017.12.108 (2018).

[7] Hao, J. J., Lu, C. X. & Li, D. H. A comparative analysis of polyacrylonitrile-based carbon fibers: (II) relationship between the microstructures and properties. *New Carbon Mater.* **35**, 802–809. 10.1016/S1872-5805(20)60528-5 (2020).

[8] Yang, F. et al. Effect of amorphous carbon on the tensile behavior of polyacrylonitrile (PAN)-based carbon fibers. *J. Mater. Sci.* **54**, 8800–8813. 10.1007/s10853-018-03256-z (2019).

[9] Li, J., Yu, Y., Li, H. & Liu, Y. Polyacrylonitrile based carbon fibers: spinning technology dependent precursor fiber structure and its successive transformation. *J. Appl. Polym. Sci.* **138**, 50988. 10.1002/app.50988 (2021).

[10] Lee, J. E. et al. Microstructural evolution of polyacrylonitrile fibers during industry-mimicking continuous stabilization. *Carbon* **195**, 165–173. 10.1016/j.carbon.2022.04.009 (2022).

[11] Furushima, Y., Nakada, M., Takahashi, H. & Ishikiriyama, K. Study of melting and crystallization behavior of polyacrylonitrile using ultrafast differential scanning calorimetry. *Polymer* **55**, 3075–3081. 10.1016/j.polymer.2014.05.015 (2014).

[12] He, J. et al. Microstructural evolution during dry-jet wet spinning postprocessing from coagulation bath fiber to high-performance polyacrylonitrile precursor fiber. *ACS Appl. Polym. Mater.* **6**, 1781–1789. 10.1021/acsapm.3c02614 (2024).

[13] Lai, C. et al. Investigation of post-spinning stretching process on morphological, structural, and mechanical properties of electrospun polyacrylonitrile copolymer nanofibers. *Polymer* **52**, 519–528. 10.1016/j.polymer.2010.11.044 (2011).

[14] Gao, Q. et al. From microfibrillar network to lamellae during the coagulation process of polyacrylonitrile fiber: visualization of intermediate structure evolution. *Macromolecules* **53**, 8663–8673. 10.1021/acs.macromol.0c01670 (2020).

[15] Gao, Q. et al. Microfibril alignment induced by stretching fields during the dry-jet wet spinning process: reinforcement on polyacrylonitrile fiber mechanical properties. *Polym. Test.* **81**, 106191. 10.1016/j.polymertesting.2019.106191 (2020).

[16] Hu, W. *Polymer Physics: A Molecular Approach* (Springer, 2012).

[17] Rubinstein, M. & Colby, R. H. *Polymer Physics* (Oxford University Press, 2003).

[18] Al Aiti, M. et al. Dry-jet wet spinning of thermally stable lignin-textile grade polyacrylonitrile fibers regenerated from chloride-based ionic liquids compounds. *Materials* **13**, 3687 (2020). 32825486 10.3390/ma13173687 PMC7504658

[19] Shin, K. A. et al. Investigation into the gelation of polyacrylonitrile solution induced by dry-jet in spinning process and its effects on diffusional process in coagulation and structural properties of carbon fibers. *Macromol. Res.* **26**, 544–551. 10.1007/s13233-018-6070-8 (2018).

[20] Wang, Y., Tong, Y., Zhang, B., Su, H. & Xu, L. Formation of surface morphology in polyacrylonitrile (PAN) fibers during wet-spinning. *J. Eng. Fibers Fabr.* **13**, 155892501801300208 (2018).

[21] Moskowitz, J. D., Jackson, M. B., Tucker, A. & Cook, J. D. Evolution of polyacrylonitrile precursor fibers and the effect of stretch profile in wet spinning. *J. Appl. Polym. Sci.* **138**, 50967. 10.1002/app.50967 (2021).

[22] Sun, L. et al. Structural changes of polyacrylonitrile fibers in the process of wet spinning. *J. Appl. Polym. Sci.* **137**, 48905. 10.1002/app.48905 (2020).

[23] Ouyang, Q. et al. Supramolecular structure of highly oriented wet-spun polyacrylonitrile fibers used in the Preparation of high-performance carbon fibers. *J. Polym. Res.* **22**, 1–10 (2015).

[24] Jiaojiao, L., Yuxiu, Y., Haojie, L. & Yaodong, L. Polyacrylonitrile based carbon fibers: spinning technology dependent precursor fiber structure and its successive transformation. *J. Appl. Polym. Sci.* **138**, 988. 10.1002/app.50988 (2021).

[25] Langford, J. I. & Wilson, A. J. C. Scherrer after Sixty years: A survey and some new results in the determination of crystallite size. *J. Appl. Crystallogr.* **11**, 102–113. 10.1107/S0021889878012844 (1978).

[26] Hassanzadeh-Tabrizi, S. A. Precise calculation of crystallite size of nanomaterials: A review. *J. Alloys Compd.* **968**, 171914. 10.1016/j.jallcom.2023.171914 (2023).

[27] Keller, A. & Kolnaar, J. *Orientational Phenomena in Polymers* 81–102 (Springer).

[28] Iqbal, O., Guo, H. & Chen, W. Structural origin of double yielding: the critical role of crystallite aggregate heterogeneity. *Macromolecules* **54**, 8381–8392 (2021).

[29] Xiong, B., Kang, J., Chen, R. & Men, Y. Initiation of cavitation upon drawing of pre-oriented polypropylene film: in situ SAXS and WAXD studies. *Polymer* **128**, 57–64. 10.1016/j.polymer.2017.09.017 (2017).

[30] Humbert, S., Lame, O., Chenal, J. M., Rochas, C. & Vigier, G. Small strain behavior of polyethylene: in situ SAXS measurements. *J. Polym. Sci., Part B: Polym. Phys.* **48**, 1535–1542. 10.1002/polb.22024 (2010).

[31] Guinier, A., Fournet, G., Walker, C. B. & Yudowitch, K. L. *Small-Angle Scattering of X-rays* (Wiley, 1955).

[32] Toki, S. et al. Entanglements and networks to strain-induced crystallization and stress–strain relations in natural rubber and synthetic polyisoprene at various temperatures. *Macromolecules* **46**, 5238–5248. 10.1021/ma400504k (2013).

[33] Luo, C. & Sommer, J. U. Frozen topology: entanglements control nucleation and crystallization in polymers. *Phys. Rev. Lett.* **112**, 195702. 10.1103/PhysRevLett.112.195702 (2014). 24877947 10.1103/PhysRevLett.112.195702

[34] Wang, Y. & Wang, S. Q. Salient features in uniaxial extension of polymer melts and solutions: progressive loss of entanglements, yielding, non-Gaussian stretching, and rupture. *Macromolecules* **44**, 5427–5435. 10.1021/ma200432q (2011).

[35] Chen, X. et al. Stretch-induced melting and recrystallization of polyethylene-plasticizer film studied by in situ X-ray scattering: A thermodynamic point of view. *J. Polym. Sci., Part B: Polym. Phys.* **56**, 1521–1528. 10.1002/polb.24740 (2018).

[36] Luo, W., Yu, Y., Wang, J. & Hu, W. Nascent structure memory erased in polymer stretching. *J. Chem. Phys.* **156**, 952. 10.1063/5.0083952 (2022). 10.1063/5.0083952 35428382

[37] Cho, B. G., Lee, J. E., Jeon, S. Y. & Chae, H. G. A study on miscibility properties of polyacrylonitrile blending films with biodegradable polymer, Shellac. *Polym. Test.* **121**, 107983. 10.1016/j.polymertesting.2023.107983 (2023).

[38] Hamedani, A. A., Ow-Yang, C. W. & Hayat Soytas, S. Silicon nanocrystals-embedded carbon nanofibers from hybrid polyacrylonitrile—TEOS precursor as high-performance lithium-ion battery anodes. *J. Alloys Compd.* **909**, 164734. 10.1016/j.jallcom.2022.164734 (2022).

[39] Chen, Y. et al. Effects of KBrO3 on chemical, aggregation and morphological structure of polyacrylonitrile (PAN) precursor fibers. *Fibers Polym.* **24**, 3007–3017. 10.1007/s12221-023-00280-y (2023).

